# Association of serum lipids with levels of leptin in hemodialysis patients

**Published:** 2013-07-01

**Authors:** Mahmoud Rafieian-Kopaei, Hamid Nasri

**Affiliations:** ^1^Medical Plants Research Center, Shahrekord University of Medical Sciences, Shahrekord, Iran; ^2^Section of Hemodialysis, Shahrekord University of Medical Sciences, Iran

**Keywords:** Leptin, Hemodialysis, Lipids, Malnutrition

## Abstract

To consider the correlation of serum leptin with dyslipidemia in hemodialysis patients. For maintenance hemodialysis patients levels of serum, lipid profiles and serum leptin were measured. Stable hemodialysis patients enrolled to the study. In all patients (36) a near significant,inverse correlation of serum leptin with serum LDL-C was seen. In male hemodialysis group a near significant positive correlation of serum leptin with serum triglyceride level was seen. The association of leptin with cholestrol and triglycerid levels could show the impact of leptin on nutrition status of hemodialysis patients.

**Implication for health policy/practice/research/medical education:**

Leptin may have an impact on serum lipids in hemodialysis patients.

## Introduction

Malnutrition is a common clinical problem in patients with end-stage kidney failure (1,2) and is associated with an increase in morbidity and mortality in haemodialysis patients (3,4). In hemodialysis , malnutrition is an independent factor causing morbidity and mortality. Serum leptin level are elevated in patients with chronic kidney insufficiency and end-stage kidney failure (5,6). Release of leptin from adipocytes may be stimulated by cytokines mediating the inflammatory response, which is frequently pronounced in patients with end-stage kidney failure on dialysis (6-8). In end-stage kidney failure, dyslipidemia is linked to risk of cardiovascular disease (9). Increased concentrations of triacylglycerol-rich, very low density lipoproteins (VLDL) and decreased concentrations of high density lipoproteins (HDL-C) are usual, while total cholesterol and low density lipoprotein (LDL-C) concentrations are not increased (9,10). In hemodialysis patients hypercholesterolemia appear to be a protective feature that are associated with a greater survival among hemodialysis patients (11). This finding is in contrast to the well-known link between over-nutrition and poor outcome in the general population (11,12). The association between under-nutrition and adverse cardiovascular outcome in hemodialysis patients, which stands in contrast to general population, has been referred to as reverse epidemiology (11-13).

## Objectives

We aimed to find a possible role for leptin on dyslipidemia in hemodialysis patients.

## Patients and Methods

### Patients

This cross-sectional study was conducted on patients on regular hemodialysis. Exclusion criteria were active or chronic infection and using NSAID or ACE inhibitors. The study was done in hemodialysis section of Shahrekord University of Medical Sciences in Shahrekord of Iran.

### Laboratory methods

After 12-hour fasting, levels of serum lipid profile containing serum triglyceride (TG ), cholestrol (Chol) and high density lipoprotein (HDL-C) level were measured using standard kits. Serum leptin (normal range of values for males is 3.84 (1.79) and for females is 7.36 (3.73 ng/ml ) was measured by enzyme-linked immunosorbent assay (ELISA) method using DRG of Germany. For the adequacy of hemodialysis the urea reduction rate (URR) was calculated from pre- and post-blood urea nitrogen (BUN) data (14). The body mass index (BMI) was calculated using weight and height (kg/m^2^) (15). Serum LDL-C was calculated using friedewald’s formula (16). Duration and dosages of hemodialysis treatment were calculated from the patients’ records. The duration of each hemodialysis session was 4 hours.

### Ethical approval

All patients signed the consent form for participation in this study. Research study was approved by the ethics committee of Shahrekord University of Medical Sciences, Iran.

### Statistical analysis

Results are expressed as the mean (SD). Comparison between the groups was done using Student’s t-test. Statistical correlations were assessed using partial correlation test. For some correlations the logarithm of data were used. Statistical analysis was performed on total hemodialysis (HD), females, males, diabetics and non-diabetics populations separately. All statistical analysis were performed using SPSS (version 11.5). Statistical significance was determined at a p< 0.05.

## Results

Of the total patients (N= 36; F= 14, M= 22 ), 26 were non-diabetic hemodialysis (F= 10, M= 16) and 10 were diabetic hemodialysis (F= 4, M= 6).

Table 1 shows patients’ data. The mean patient age was 46 (16) years. The mean length of the time they received hemodialysis was 30 (36) (median: 17.5) months. The mean serum leptin was 7 (9.2) (median: 4.2) ng/ml. The mean serum leptin values within the diabetic and non-diabetic groups were 7.63 (4.63) (median: 7.7) and 6.85 (10.47) (median: 3.45) ng/ml, respectively. The mean serum cholestrol level of all the patients was 118 (39) mg/dl. The mean serum cholestrol values with in the diabetic and non-diabetic groups were 136 (50) and 111 (32) mg/dl, respectively. In this study no significant difference of serum leptin between males and females of non-diabetic hemodialysis was detected. However a significant difference of serum leptin between males and females of diabetic hemodialysis was seen (r= 0.035). A significant inverse correlation of serum Chol with hemodialysis efficacy (r= -0.38, p= 0.024) was seen too. In all patients a near significant inverse correlation of serum leptin with serum LDL-C (r= -0.29, p= 0.09) and a near significant correlation of serum Tg with logarithm of serum leptin (r= 0.30, p= 0.078) were seen too (adjusted for age). In male hemodialysis group a significant positive correlation of serum leptin with duration of hemodialysis (r= 0.45, p= 0.035) (adjusted for age) and a significant positive correlation of serum leptin with ages of the patients (r= 0.44, p= 0.046) (adjusted for dialysis dosage) was seen too. In this group also a near significant positive correlation of serum leptin with serum triglyceride level (r= 0.42, p= 0.06) (adjusted for age) was seen.

**Table 1:** Patients data

**Total patients** **n=36**	**Minimum**	**Maximum**	**Mean±SD**	**Median**
Age (years)	16	80	46±16	43
URR (%)	39	76	59±9	57.5
Leptin (ng/ml)	0.1	52	7±9.1	4.2
Chol (mg/dl)	59	211	117±39	115.5
TG (mg/dl)	29	461	130±95	94
LDL (mg/dl)	12	122	61.6±22	36.5
HDL (mg/dl)	20	70	38±11	60
Non diabetics (n=26)				
Age (years)	16	80	43.6±16.5	41.5
URR (%)	50	76	60.6±7.78	60
Leptin (ng/ml)	0.1	52	6.8 ± 10	3.45
Chol (mg/dl)	59	171	111 ± 32	115
TG (mg/dl)	61	461	128± 84	97
LDL (mg/dl)	12	99	62 ± 20	36
HDL (mg/dl)	25	70	39 ± 11	60
Diabetics (n=10)				
Age (years)	27	75	51±16	55
URR (%)	39	75	54±10	54
Leptin (ng/ml)	0.2	15	7.6±4.6	7.8
Chol (mg/dl)	60	211	136±50	130
TG (mg/dl)	29	456	136±126	88.5
LDL (mg/dl)	26	122	62± 27	38.5
HDL (mg/dl)	20	46	35±9.8	58

## Discussion

In this study we found a near significant inverse correlation of serum leptin with serum LDL-C. In male hemodialysis group a significant positive correlation of serum leptin with hemodialysis duration and also with the ages of the patients was seen. In this group also a near significant positive correlation of serum leptin with serum triglyceride level was seen. In the study conducted by Fox *et al.* on 812 incident hemodialysis found a 40% prevalence of hyperlipidemia in patients (4). To compare 46 hemodialysis patients with 56 healthy subjects in the aspect of serum lipids, Gillett *et al.* found, total, and LDL-C were unchanged, triacylglycerols and free cholesterol were raised and HDL-C concentrations were significantly decreased compared to controls (10). Previously, we showed that on thirty-six patients with the mean of ages of 47.5 years old, the mean cholestrol level was 153.4 (31.3) mg/dl, also the mean triglyceride level was 135.1 (66.1) mg/dl (9). Concerning these results, studies showed that cholesterol levels is inversely associated with mortality in dialysis patients. This paradox may be explained by systemic inflammation and/or malnutrition, which are associated with lower cholesterol concentration and higher mortality (17). Indeed hemodialysis patients have a high risk of atherosclerotic cardiovascular disease, but dialysis patients with higher serum cholesterol have lower mortality rates (4). Recent epidemiologic studies have shown that increased serum value of leptin may reduce nutrient intake and contribute to the development of protein-energy malnutrition (7) that may be shown by low levels of serum cholestrol. In a study on 52 hemodialyzed individuals, Zbroch *et al.* found that serum lipid concentrations had no correlation with leptin (18). In another study conducted by Bossola* et al.* on 24 healthy subjects and in 49 end-stage kidney disease on maintenance hemodialysis, no significant correlation between serum leptin level and cholesterol was found (19). In an accord with our findings, Obineche *et al.* measured serum leptin levels in 150 patients on hemodialysis, peritoneal dialysis or in the predialysis phase of chronic renal failure, and observed significantly elevated levels of leptin, particularly in female patients, and leptin was shown to correlate significantly with total and LDL-C (20). To compare serum levels of leptin, some nutritional parameters and serum lipids in hemodialyzed patients (n= 46) and healthy subjects (n= 24) and to explore the association of serum leptin level and the nutritional parameters in both groups, Svobodova *et al*, detected, firstly the low serum triglyceride level compared to controls and secondly serum leptin level in hemodialysis, correlated positively with serum cholesterol and triglyceride levels (21). Inverse correlations of serum cholestrol with duration and dosage of hemodialysis and with dialysis adequacy as well, may shows the effects of poorly adequate dialysis on exaggerating the malnutrition and also dyslipidemia of hemodialysis patients.

## Conclusion

Our data supports the impact of leptin on cholestrol and triglycerid levels.

## Authors’ contributions

MRK and HN wrote the manuscript equally.

## Conflict of interests

The authors declared no competing interests.

## Ethical considerations

Ethical issues (including plagiarism, misconduct, data fabrication, falsification, double publication or submission, redundancy) have been completely observed by the authors.

## Funding/Support

This study was supported by a grant from Shahrekord University of Medical Sciences.
